# Type 2 diabetes associated variants of *KCNQ1* strongly confer the risk of cardiovascular disease among the Saudi Arabian population

**DOI:** 10.1590/1678-4685-GMB-2017-0005

**Published:** 2017-08-31

**Authors:** Maha S. Al-Shammari, Rhaya Al-Ali, Nader Al-Balawi, Mansour S. Al-Enazi, Ali A. Al-Muraikhi, Fadi N. Busaleh, Ali S. Al-Sahwan, Abdulmohsen Al-Elq, Awatif N. Al-Nafaie, Jesu Francis Borgio, Sayed AbdulAzeez, Amein Al-Ali, Sadananda Acharya

**Affiliations:** 1 College of Medicine, University of Dammam, Kingdom of Saudi Arabia; 2 King Fahd Hospital of the University, University of Dammam, Dammam, Kingdom of Saudi Arabia; 3 Institute for Research and Medical Consultations (IRMC), University of Dammam, Kingdom of Saudi Arabia

**Keywords:** T2D, CVD, KCNQ1, genetic association, Saudi population

## Abstract

Genome-wide association studies have identified several loci associated with an increased risk for cardiovascular disease (CVD) and type 2 diabetes (T2D). Polymorphisms within the *KCNQ1* (potassium voltage-gated channel, KQT-like subfamily, member 1) gene are consistently associated with T2D in a number of populations. The current study was undertaken to evaluate the association of 3 polymorphisms of KCNQ1 (rs2237892, rs151290 and rs2237895) with T2D and/or CVD. Patients diagnosed with either T2D (320 patients), CVD (250 patients) or both (60 patients) and 516 healthy controls were genotyped by TaqMan assay run on a real time PCR thermocycler. A statistically significant association was found for SNPs rs151290 (OR = 1.76; 95%CI = 1.02-3.05; p = 0.0435) and rs2237895 (OR = 2.49; 95%CI = 1.72-3.61; p < 0.0001) with CVD. SNP rs151290 (OR = 7.43; 95%CI = 1.00-55.22; p = 0.0499) showed a strong association in patients with both T2D and CVD. None of the SNPs showed any significant association with T2D. Haploview analysis showed that the ACC (rs151290, rs2237892 and rs2237895) haplotype is the most significant risk allele combination for CVD, while CCA is the most significant risk haplotype for co-morbidity with T2D. *KCNQ1* polymorphism at SNPs rs151290 and rs2237895 is strongly associated with CVD in this population, but presented no association with T2D.

## Introduction

Independent genome-wide association studies (GWAS) have greatly advanced the genetics of common diseases, including cardiovascular diseases (CVD), diabetes, and cancer. Presently, over 4,800 independent loci have Hajjar reported to be associated with different diseases in various populations. Recently, a number of GWAS studies have reported a strong association between *KCNQ1* (potassium voltage-gated channel, KQT-like subfamily, member 1) and type 2 diabetes mellitus (T2D). This association has been reported in various ethnic populations, including a number of European and Scandinavian countries (Unoki *et al.*, 2008; Jonsson *et al.*, 2009) in addition to Asian countries, specifically China (Hu *et al.*, 2009; Liu *et al.*, 2009; Qian *et al.*, 2015), Korea (Lee *et al.*, 2008), Japan (Yasuda *et al.*, 2008), Germany (Mussig *et al.*, 2009) and India (Been *et al.*, 2011). The *KCNQ1* gene, which is located on chromosome 11q, encodes a protein that is involved in cardiac action potential. Hence, mutations in this gene are expected to influence cardiac function and are associated with the development of a number of diseases.

The Eastern Province of Saudi Arabia is known for its high prevalence of T2D and CVD. This imposes a tremendous economic and social burden on the region's population. As far as we are aware, no genetic study has been conducted on this population group with regard to the association of *KCNQ1* with T2D or CVD. Therefore, the present study was conducted to determine whether *KCNQ1* SNPs at loci rs151290, rs2237892 and rs2237895 are associated with T2D and CVD in the Saudi population of the Eastern Province. These particular SNPs were selected as they are more frequently reported as having an association with T2D in other populations.

## Material and Methods

### Study subjects

A total of 640 Saudi patients comprising 330 individuals who had been clinically diagnosed with T2D, 250 with CVD and 60 with both T2D and CVD were recruited for the study. Additionally, the study group included 516 subjects recruited at the blood bank of King Fahd Hospital of the University after confirmation that the donors were free from T2D, CVD or any family history of these two conditions. T2D was confirmed by the definitions of the World Health Organization, 2006, while CVD cases were confirmed by a history of one or more myocardial infarction (MI). The study was approved by the University of Dammam Institutional Review Board (IRB-2013-08-026) and written informed consent was obtained from all participating subjects.

### Sample collection and DNA analysis

Five milliliters of peripheral blood was collected from each participant by venipuncture into a heparinized vacutainer tube (BD Diagnostics, USA). DNA was extracted using commercially available genomic DNA purification kits (GE Healthcare, Buckinghamshire, United Kingdom). DNA concentrations and purity were measured and confirmed in a NanoDrop2000 spectrophotometer (Thermo Fisher Scientific Inc., Massachusetts, USA). The extracted DNA was stored at −80 °C for further analysis.

### Genotyping

Genotyping of the *KCNQ1* SNPs, at loci rs151290, rs2237892 and rs2237895 was performed by TaqMan assay (Applied Biosystems, Foster City, CA, USA). The reactions (25 μL) were run in a 96 well PCR plate on a real time PCR thermocycler (7500 Fast, ABI, Foster City, CA, USA) as per the manufacturer's recommendations. The final end-point allele calling was determined using ABI software 7500 version. 2.0.6. About 5% of the samples were run for genotyping quality assessment and were confirmed for concordance with the primary genotyping assay.

### Statistical analysis

The biographic, hematological and biochemical parameters were reported as means ± standard deviation. Intergroup significance for each variable was calculated by either Chi-square or Student's *t*-test using SPSS software. Logistic regression was used to estimate the OR (odds ratio) between the groups at 95% CI. Data were stratified into four clusters, namely T2D only, T2D+CVD, CVD only, and controls to analyze the association by logistic regression. All three SNPs were tested for Hardy-Weinberg equilibrium (HWE) using a Chi-square test. HaploView ver.4.2 was used to analyze linkage disequilibrium (LD) and three loci risk haplotypes (Barrett *et al.*, 2005). Test values were considered significant when p < 0.05.

## Results

The biometric parameters obtained from the patient and control individuals are given in Table 1. Allele and genotype frequencies for the SNPs tested are shown in Table 2. The male and female representation in the sample is skewed towards the male group, which may be attributed to participation bias. No significant difference in mean age among the various groups was found. There were significant differences between diabetic and non-diabetic groups for BMI and blood glucose level.

**Table 1:** Description of type 2 diabetes (T2D), cardiovascular disease (CVD) and control subjects that participated in the study.

Parameter	Control subjects (n = 516)	T2D patients (n = 320)	CVD patients (n = 250)	T2D + CVD patients (n = 60)
Male (%)	289 (56.01)	174 (54.38)	163 (65.20)	48
Female (%)	227 (43.99) p < 0.0060	146 (45.63) p = 0.1175	87 (34.80) p < 0.0001	12 p < 0.0001
Mean age (years)	48.75 ± 6.85	51.50 ± 8.75 p < 0.8875	52.3 ± 13.8 p < 0.8870	54 ± 9.45 p < 0.7783
Age range (years)	25-65	28-82	32-85	42-78
BMI (kg/m^2^)	25 ± 3.8	27.2 ± 4.6 p < 0.0001	32.59 ± 5.3 p < 0.0001	33.47 ± 6.5 p < 0.0001
Glucose (mmol/L)	4.92 ± 3.8	8.15 ± 4.2 p < 0.0001	5.22 ± 5.6 p < 0.3837	8.27 ± 3.6 p < 0.0001

BMI: body mass index. *p < 0.05 was considered significant.

**Table 2:** Genotype analysis relating to *KCNQ1* variants and risk of type 2 diabetes (T2D), cardiovascular disease (CVD) and T2D+CVD comorbidity.

SNP variants of *KCNQ1*	Allele / Genotype	Control subjects (n = 516)	T2D			CVD			T2D + CVD		
			Patients (n = 330)	OR (95% CI)	p-value*	Patients (n = 250)	OR (95% CI)	p-value*	Patients (n = 60)	OR (95% CI)	*p*-value*
rs151290 (A > C)	A allele	275 (26.65)	185 (28.03)	-	-	184 (37.25)	-	**-**	17 (14.17)	-	**-**
	C allele	757 (73.35)	475 (71.97)	0.9327 (0.7494-1.1609)	0.5328	310 (62.75)	0.6120 (0.4870-0.7693)	**< 0.0001**	103 (85.83)	2.2010 (1.2938-3.7443)	**0.0036**
	AA	49 (9.50)	32 (9.70)	-	-	22 (8.91)	-	-	1 (1.67)	-	**-**
	AC	177 (34.30)	121 (36.67)	1.0468 (0.6337-1.7291)	0.8583	140 (56.68)	1.7617 (1.0167-3.0525)	**0.0435**	15 (25.0)	4.1525 (0.5352-32.2197)	0.1732
	CC	290 (56.20)	177 (53.63)	0.9346 (0.5765-1.5151)	0.7838	85 (34.41)	0.6528 (0.3736-1.1407)	0.1342	44 (73.33)	7.4345 (1.0010-55.2165)	**0.0499**
rs2237892 (C > T)	C allele	1007 (97.58)	647 (98.03)	-	-	484 (97.58)	-	-	120 (100)	-	-
	T allele	25 (2.42)	13 (1.97)	0.8093 (0.4111-1.5934)	0.5405	12 (2.42)	0.9987 (0.4975-2.0047)	0.9970	0 (0)	0.1639 (0.0099-2.7102)	0.2064
	CC	496 (97.12)	319 (96.67)	-	-	236 (95.16)	-	-	60 (100)	-	-
	CT	15 (2.91)	9 (2.73)	0.9329 (0.4034-2.1573)	0.8710	12 (4.84)	1.6814 (0.7748-3.6487)	0.1887	0 (0)	0.2647 (0.0156-4.4807)	0.3571
	TT	5 (0.97)	2 (0.61)	0.6219 (0.1199-3.2251)	0.5717	0 (0)	0.1909 (0.0105-3.4660)	0.2628	0 (0)	0.7461 (0.0407-13.6595)	0.8434
rs2237895 (A > C)	A allele	627 (60.76)	394 (59.70)	-	-	237 (47.59)	-	**-**	85 (70.83)	-	**-**
	C allele	405 (39.24)	266 (40.30)	1.0452 (0.8562-1.2760)	0.6641	261 (52.41)	1.7049 (1.3740-2.1154)	**< 0.0001**	35 (29.17)	0.6375 (0.4218-0.9634)	**0.0326**
	AA	202 (39.15)	122 (36.97)	-	-	97 (38.96)	-	-	31 (51.67)	-	-
	AC	223 (43.22)	150 (45.45)	1.1137 (0.8205-1.5117)	0.4896	43 (17.27)	0.4016 (0.2674-0.6029)	**< 0.0001**	23 (38.33)	0.6721 (0.3793-1.1908)	0.1733
	CC	91 (17.63)	58 (17.58)	1.0553 (0.7083-1.5723)	0.7913	109 (43.77)	2.4944 (1.7249-3.6072)	**< 0.0001**	6 (10.0)	0.4296 (0.1732-1.0658)	0.0684

Values in parenthesis are percentages or 95% C.I.

* p < 0.05 was considered significant

None of the *KCNQ1* SNPs tested showed a statistically significant association with T2D. We further stratified the samples into T2D+CVD group for the analysis. Interestingly, comorbidity with T2D+CVD patients showed a statistically significant association for SNP rs151290 with the homozygous mutant risk genotype (OR = 7.4345; p = 0.0499), whereas two other SNPs (rs2237892 and rs2237895) did not show an association.

Additional recruitment of 250 more CVD patients to the sample and stratified data analysis further revealed that SNPs rs151290 with heterozygous mutant risk genotype (OR = 1.7617; p = 0.0435) and rs2237895 with homozygous mutant risk genotype (OR = 2.4944; p < 0.0001) are strongly associated with the risk of developing CVD among the Eastern Province Saudi population.

**Figure 1:**
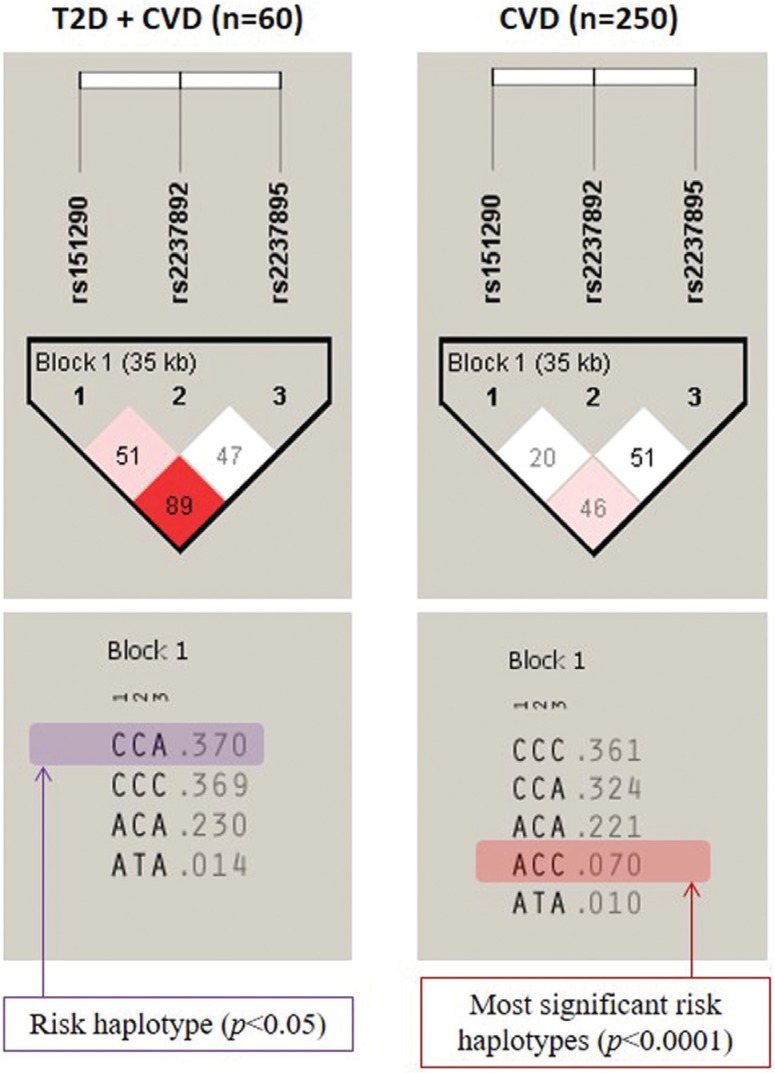
HaploView analysis of *KCNQ1* SNPs; block 1 comprises SNPs rs151290, rs2237892 and rs2237895 (35kb). Red or pink block, D' (linkage disequilibrium) < 1.0, with LOD score > 2.0; white blocks, D' < 1.0 with LOD score < 2.0

Haplotype analysis (Figure 1) revealed that ACC haplotypes (in the order of rs151290, rs2237892 and rs2237895) show the most significant risk (p < 0.0001) allele combinations for CVD, while CCA is the most significant comorbidity risk haplotype for T2D and CVD.

## Discussion

The Human Genome Project has demonstrated the important role that genetic polymorphisms play in the susceptibility and severity of both CVD and T2D. Specific polymorphisms have been recognized as being associated with an increased risk of both these diseases. Studies have identified several polymorphisms (within *KCNQ1*) that are most commonly found in different populations (Lee *et al.*, 2008; Unoki *et al.*, 2008; Yasuda *et al.*, 2008; Jonsson *et al.*, 2009; Hu *et al.*, 2009; Liu *et al.*, 2009; Mussig *et al.*, 2009; Been *et al.*, 2011; Qian *et al.*, 2015), albeit with differing frequencies. However, some reports have found a weak association between T2D and these polymorphisms.

Our study is reporting, for the first time in any population, an association between *KCNQ1* SNPs rs151290 and CVD. We are also reporting an association between *KCNQ1* SNPs rs151290 in T2D Saudi patients with CVD. There are reports that the mutant *KCNQ1* is implicated in various cardiac dysfunctions caused by lipid metabolism (Chen *et al.*, 2010), familial atrial fibrillation (Bartos *et al.*, 2013), and long QT syndrome (Hajjar and Hulot, 2014). Our findings may support those reports that these polymorphisms in *KCNQ1* could lead to an increased risk of developing CVD. Our data also indicate that there is no statistically significant association between these SNPs in *KCNQ1* and patients with T2D. Our findings are in line with a study by Bazzi *et al.* (2014) that also reported a lack of association between *KCNQ1* SNP rs2237892 in the Saudi population with T2D. Furthermore, a study conducted on North African Arabs also revealed a lack of association between SNPs in *KCNQ1* and T2D (Turki *et al.*, 2012). Our data contradict other studies which have suggested that these SNPs are strongly associated with T2D (Lee *et al.*, 2008; Unoki *et al.*, 2008; Yasuda *et al.*, 2008; Jonsson *et al.*, 2009; Hu *et al.*, 2009; Liu et *al*., 2009; Mussig *et al.*, 2009; Been *et al.*, 2011; Qian *et al.*, 2015).

Haplotype analysis of all three SNPs showed a significant risk factor for CVD [haplotype ACC: rs151290, rs2237892 and rs2237895], which confirms the allele and genotype analysis, while the CCA haplotype is a risk haplotype for T2D patients with CVD.

These results indicate that *KCNQ1* polymorphism plays a significant role in the development of CVD in the Saudi population. However, the association of polymorphisms in *KCNQ1* with T2D is ethnically specific and does not seem to contribute towards the development of T2D in the Saudi population. Our results implicate *KCNQ1* variants to be strongly associated with the risk of development of CVD, which is reported here for the first time for any population.

## Conclusion

*KCNQ1* gene polymorphism at loci rs151290 and rs2237895 is strongly associated with CVD, while rs151290 is associated with comorbidity of CVD and T2D in the Saudi population of the Eastern Province. However, these variants, in addition to rs2237892, have no association with T2D in this population.
